# Does bone preparation impact its shape: consequences for comparative analyses of bone shape

**DOI:** 10.7717/peerj.7932

**Published:** 2019-11-28

**Authors:** Fanny Pagès, Anne-Claire Fabre, Anick Abourachid

**Affiliations:** 1 UMR 7179 C.N.R.S/M.N.H.N., Mécanismes adaptatifs et évolution (MECADEV), Muséum National d’Histoire Naturelle, Paris, France; 2 Life Sciences Department, The Natural History Museum, London, UK

**Keywords:** Osteological preparation, Bone deformation, Geometric morphometrics, Intraspecific and Interspecific analyses

## Abstract

Vertebrate osteological collections provide comparative material for morphological analysis. Before being stored in the collection and studied by researchers, specimens are treated by preparators or curators and are cleaned. The preparation protocol employed ideally should not damage the material. Here, we explore the potential deformation of bones due to preparation using geometric morphometric methods. We focus both on intraspecific and interspecific variability. Our data on the scapular girdle of birds show that, at an intraspecific level, the effect of preparation on bone shape cannot be neglected. Paired and unpaired bones did not respond to the preparation process in the same way, possibly due to differences in function and their anatomical characteristics. Moreover, deformations due to preparation can be estimated by looking at the texture of the bone. At the interspecific level, we found no significant differences as the deformations induced by preparation are relatively small compared to differences among species. This study highlights the importance of carefully selecting preparation methods in order to avoid physical damage that could impact the shape of bones, especially for studies at the intraspecific level.

## Introduction

Museum collections provide a rich source of anatomical material, often collected over the span of several centuries. These collections provide access to specimens, allowing for the study of a broad diversity and large number of animals from around the world. Before being added to collections, specimens are usually treated by preparators or curators. In order to prepare osteological material, common before the advent of computed microtomography facilities, specimens have to be cleaned using either natural (ranging from natural maceration, cleaning by boiling, to cleaning by bugs such as terrestrial isopods, marine isopods or dermestid beetles) or chemical (enzyme detergent soup, hydrogen peroxide or potassium hydroxide) treatments. Next, bones are dried using different techniques (natural drying lying on a flat surface or dried with artificial heat) allowing access to the bones (Fernández-Jalvo & Marin-Monfort, 2008). In theory, the preparation methods employed should not damage the integrity of the material. Thus, the protocol used should be adapted with products that are compatible with the material treated and must not interfere with possible future scientific studies. Possible consequences on the integrity of different skeletal elements depending on the preparation protocol used have already been studied and reported in several papers (Fernández-Jalvo & Marin-Monfort, 2008; Hahn, Vogel & Delling, 1991; Lemoine, 2011). Such consequences can be somehow compared to morphological deformations induced by the processes of fossilization (i.e., taphonomy). Only few studies have attempted to characterize taphonomical processes and to develop approaches taking into account the deformation induced by these taphonomic effects (Denys, 2002; Fernández-Jalvo & Andrews, 2016; Lyman, 2010). Indeed, the consequences of preparation on bones often remain underestimated and poorly documented (López-Polín, 2012). However, a study of Fernández-Jalvo & Marin-Monfort (2008) evaluated the effect of preparation methods on bones using electron microscopy. They found that for a same bone, only 2 out of the 12 methods used could be recommended: burying and the use of enzymes with close control of the duration to minimize damage. Furthermore, another method was acceptable but not excellent: the use of potassium hydroxide (KOH) with careful control of the duration to avoid the risk of damage. This study highlights the importance of carefully selecting the preparation method in order to avoid physical damage that could impact the structure and shape of the treated bones.

Here, we decided to investigate variation in bone shape due to preparation given the large amount of variability observed in collection specimens. We predict that these deformations could be due to the preparation process using chemicals dissolving fat and proteins. However, some parts of the bone may also be more easily deformed (Fernández-Jalvo & Marin-Monfort, 2008; Hahn, Vogel & Delling, 1991). We further predict that these deformations can have an impact on morphometric studies. Preparation deformations can cause and render more complex intra-individual and intra-species variability, modifying the bone shape depending of its composition, function, thickness or robustness (Lemoine, 2011).

**Figure 1:**
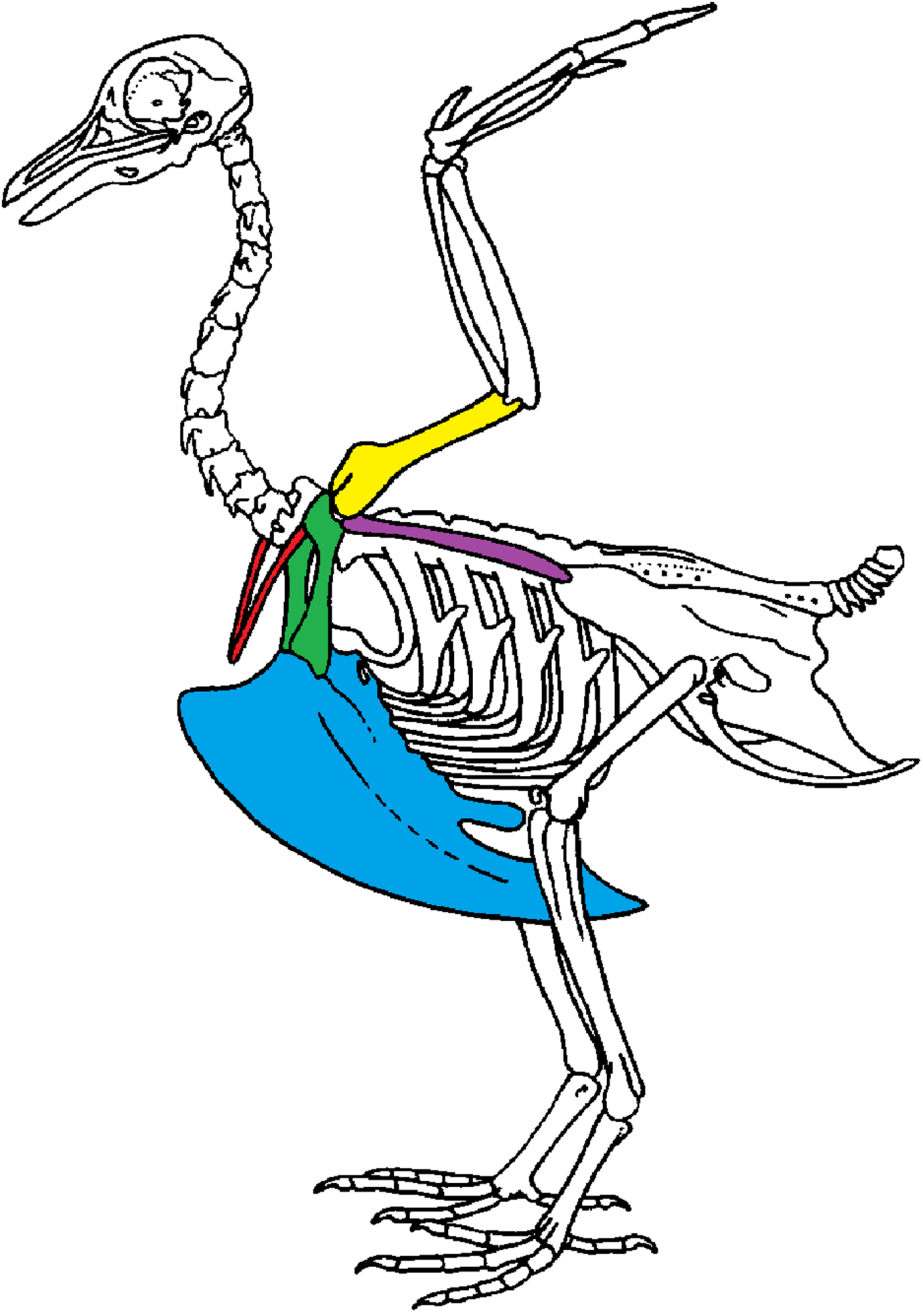
Scapular girdle of bird. Drawing of a complete bird skeleton with scapular bones of interest highlighted in colors: furcula in red, sternum in blue, coracoids in green, scapula in purple, humerus in yellow. Modified from BIODIDAC.

We use geometric morphometric methods as these methods are commonly used to detect shape differences and are sensitive to small variations in shape. Shape variability can either be natural (natural variability including variability due to the functional role of a bone) or non-natural (due to preparation). We focused on the bones of the scapular girdle in birds. The scapular girdle is composed by two unpaired bones: the sternum and the furcula, and three paired bones: the scapula, the coracoid and the humerus (Fig. 1). All these bones have an important role during locomotion, as muscles involved in wing movements are attached to them.

Two types of functions in scapular girdle of birds were identified previously: (1) bones that need to resist the action of the muscles attached and that thus need to be robust and (2) bones that play a role of protection of the internal organs such as the heart and viscera. Both of these functions may also be related to bone flexibility, like the spring function of the furcula, which can absorb and return energy during the wingbeat (Goslow, Dial & Jenkins, 1989; Kardong, 2012; Mitchell et al., 2017).

To assess the impact of preparation on bone, we analyzed the texture of the bone, its shape variation (disparity), and its asymmetry. The asymmetry was defined as significant differences in shape within a single specimen. We expect that the asymmetry should be higher if there is a preparation effect. To assess whether the observed deformations may impact subsequent analyses, we compared effects at the intraspecific and interspecific level.

## Materials and Methods

### Material

We sampled 20 complete quail skeletons (*Coturnix coturnix*, Galliformes). These quail bones are housed in the research collection of A. Abourachid. All specimens were bred in captivity and prepared using the same protocol (see protocol below). In order to assess whether the intraspecific variability is lower than the interspecific one, we added several other species. We sampled one individual of six species from the collections of the Museum National d’Histoire Naturelle (Paris, France). Four are closely related to quails: *Meleagris gallopavo* (Galliformes), *Anseranas semipalmata*, *Chauna chavaria* and *Cygnus olor* (Anseriformes, sister group) and two share the same flight type: *Coua cristata* (Cuculiformes) and *Cariama cristata* (Gruiformes) (Table 1). We selected one individual per species for the interspecific dataset.

**Table 1:** Details of specimens used in analyses. Detailed family, order, species name and number of individuals included (*N*).

Family	Order	Species	Collection code	*N*
Anseriformes	Anatidae	*Cygnus olor*	MNHN-ZO-1871-420	1
Anseriformes	Anseranatidae	*Anseranas semipalma*	MNHN-ZO-2004-151	1
Anseriformes	Anhimidae	*Chauna chavaria*	MNHN-ZO-1921-255	1
Galliformes	Phasianidae	*Meleagris gallopavo*	MNHN-ZO-1873-174	1
Cuculiformes	Cuculidae	*Coua cristata pyropyga*	MNHN-ZO-1883-517	1
Gruiformes	Cariamidae	*Cariama cristata*	MNHN-ZO-1934-614	1
Galliformes	Phasianidae	*Coturnix coturnix*	Abourachid’s scientific collection	20

### Preparation protocol

The preparation protocol used for the quail data set is composed of ten steps. First, the birds are eviscerated and feathers, skin and viscera are removed. Then, large muscles are removed (defleshing). This is facilitated by carcass reduction (dismemberment and decapitation). Carcasses are then boiled for three hours and put into a lukewarm salt water bath with an addition of an enzyme (papain: cysteine protease enzyme; 1 g/L) for 48 h at 60 °C. At the end of this step, the bones are put into a lukewarm sodium perborate bath until chilled (for more than 24 h). At that point, bones are well separated and free of flesh. Bones are rinsed and dried, lying on an absorbent surface (for 24 h). Finally, if after drying step, traces of fat persist on the surface of the bones they are put in a bath of absolute alcohol for several days and the renewal of the bath is possible many times according to the state of saturation in bone fat (yellowish coloration). When bones appear no longer saturated a final drying step is necessary to evaporate the alcohol.

### 3D data collection

We generated 3D surface scans with a white light fringe Breuckmann scanner (SmartSCAN) and its scanning software Optocat (http://gmv.cast.uark.edu/scanning-2/software/opticat/) at the “plateforme de morphométrie” of the UMS 2700 of the MNHN. The scanner consists of a projector of white light fringes and two cameras that are positioned asymmetrically around the projector. Data on the surface of a bone are accurately captured and reconstructed by triangulation angles implemented in the Optocat software. It finally produces a high-resolution meshed 3D object which provides a representation of the surface of the bone only. For each specimen, we scanned eight bones: a sternum, a furcula, both coracoids (right and left), both scapulae (right and left) and both humeri (right and left). Further processing is performed with the Geomagic Studio 2013 (http://www.geomagic.com) software package in order to obtain a surface on which data can be accurately acquired.

## Methods

### Shape quantification using geometric morphometric

In order to assess the effect of the deformations of the bone and its potential effect on shape analysis, we use 3D geometric morphometric analysis on our sample of seven species of birds. Geometric morphometrics allow a quantification of shape variation using Cartesian landmark coordinates. This approach permits to quantitatively study the shape variation of bones in relation to quantitative and qualitative traits. We created as set of landmarks in order to quantify morphological disparity.

Morphometric data were acquired on each surface scan of each bone using the IDAV Landmark software. For each bone, landmarks were chosen to accurately describe the complex geometry of each element. We used anatomical landmarks as well as sliding semi landmarks of curves and on surfaces to describe bone shape more accurately. Anatomical landmarks and sliding semi landmarks of curves were manually acquired on each scan by the same person (F.P.) whereas sliding semilandmarks on surfaces were semi-automatically projected onto the surface of each bone using the approach described below (see 3D sliding-landmarks procedures). To be able to compare the paired bones, we mirrored right bones into left bones, allowing to include all paired bones in the same comparative analysis. We kept the side information for each paired bone.

**Figure 2:**
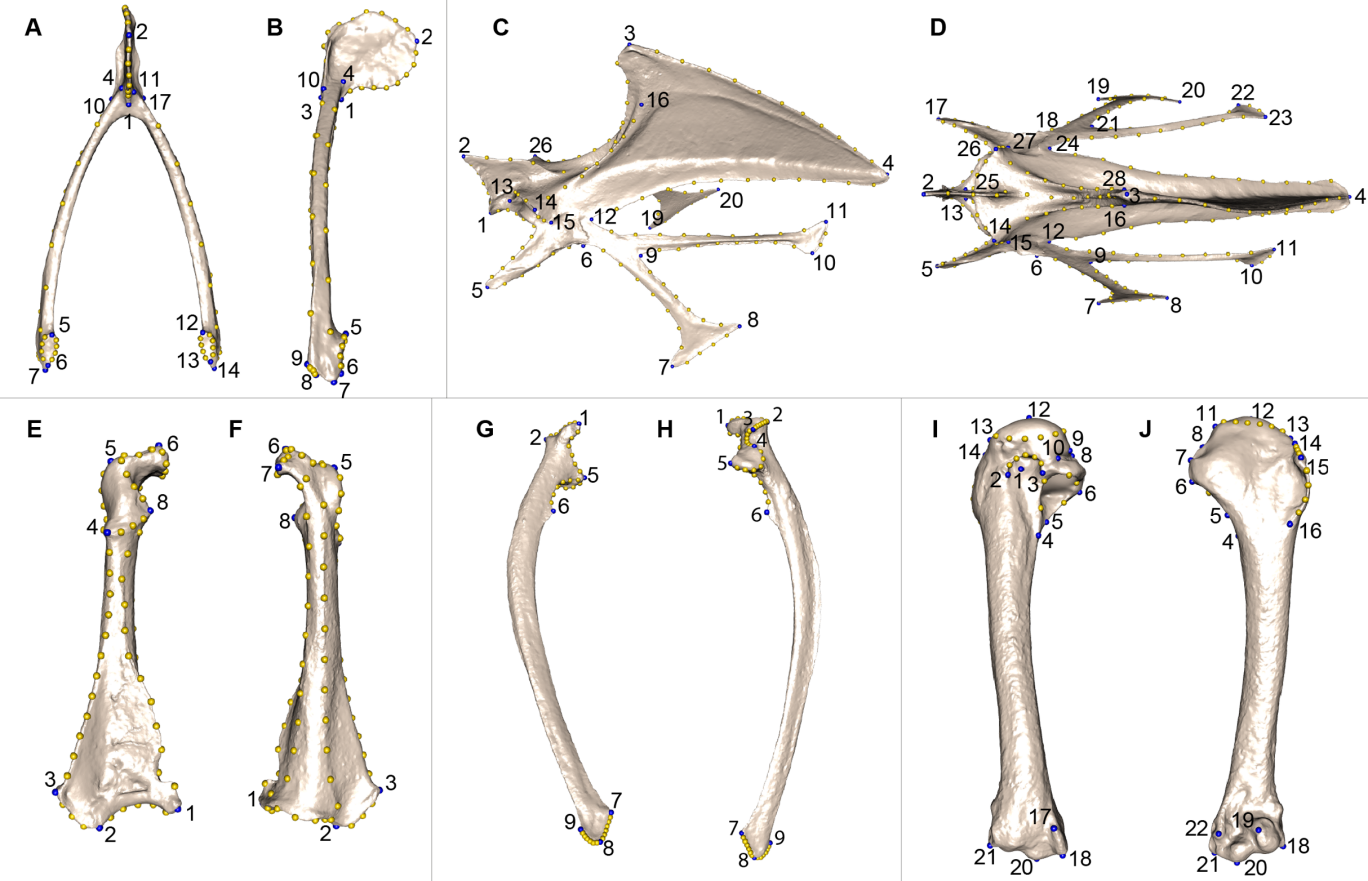
Landmarks used in the analyses to quantify shape variation on scapular bones. Quail bones are presented. Furcula: (A) caudal view, (B) lateral view, see Table 2 for landmark definition. Sternum: (C) lateral view, (D) ventral view, see Table 3 for landmark definitions. Left coracoid: (E) dorsal view, (F) ventral view, see Table 4 for landmark definitions. Left scapula: (G) dorsal view, (H) ventral view, see Table 5 for landmark definitions. Left humerus: (I) medial view, (J) lateral view, see Table 6 for landmark definitions. Blue points represent landmarks and gold points represent semi-landmark curves.

We defined a unique set of landmarks and curves for each bone. Furculae are described using 814 points (17 landmarks, 70 curve points and 727 surface points; Table 2; Figs. 2A and 2B), the sternum shape with 3,738 points (28 landmarks, 176 curve points and 3,534 surface points; Table 3; Figs. 2C and 2D), the coracoids with 1,080 points (8 landmarks, 87 curve points and 985 surface points; Table 4; Figs. 2E and 2F), the scapulae with 744 points (7 landmarks, 47 curve points and 690 surface points; Table 5; Figs. 2G and 2H) and the humeri with 813 points (22 landmarks, 29 curve points and 762 surface points; Table 6; Figs. 2I and 2J).

**Table 2:** Definition of the landmarks of the furcula used in the geometric morphometric analysis. See Figs. 2A and 2B for landmark position on the furcula.

Landmarks	Definition
1	Dorsal extremity of the symphysis, cranial view
2	Ventral extremity of the symphysis, caudal view
3	Fusion point of the two clavicles
4	Ventral point of the clavicle and symphysis fusion, right clavicle
5	Rostral extremity of the acrocoracoidal articular facet, right clavicle
6	Caudal extremity of the acrocoracoidal articular facet, right clavicle
7	Most caudal point of the right clavicle
8	Caudal extremity of the acromialis process, right clavicle
9	Rostral caudal extremity of the acromialis process, right clavicle
10	Dorsal point of the clavicle and symphysis fusion, right clavicle
11	Ventral point of the clavicle and symphysis fusion, left clavicle
12	Rostral extremity of the acrocoracoidal articular facet, left clavicle
13	Caudal extremity of the acrocoracoidal articular facet, left clavicle
14	Most caudal point of the left clavicle
15	Caudal extremity of the acromialis process, left clavicle
16	Rostral caudal extremity of the acromialis process, left clavicle
17	Dorsal point of the clavicle and symphysis fusion, left clavicle

**Table 3:** Definition of the landmarks of the sternum used in the geometric morphometric analysis. See Figs. 2C and 2D for landmark position on the sternum.

Landmarks	Definition
1	Cranial extremity of the dorsal manubrial spine
2	Craniodorsal extremity of the manubrium
3	Dorsal extremity of the cranial process of the keel
4	Caudal extremity of the caudal process of the keel body
5	Cranial extremity of the craniolateral process, left side
6	Caudal extremity of the last sternal rib facet, left side
7	Dorsal extremity of the first trabecula, left side
8	Ventral extremity of the first trabecula, left side
9	Junction point of the two trabeculae, left side
10	Dorsal extremity of the second trabecula, left side
11	Ventral extremity of the second trabecula, left side
12	Junction point of the second trabecula and the sternum body, left side
13	Medial extremity of the dorsolateral process of coracoidal articular facet, left side
14	Ventral extremity of the dorsolateral process of coracoidal articular facet, left side
15	Lateral extremity of the dorsolateral process of coracoidal articular facet, left side
16	Ventral extremity of the lateral crest, left side
17	Cranial extremity of the craniolateral process, right side
18	Caudal extremity of the last sternal rib facet, right side
19	Dorsal extremity of the first trabecula, right side
20	Ventral extremity of the first trabecula, right side
21	Junction point of the two trabeculae, right side
22	Dorsal extremity of the second trabecula, right side
23	Ventral extremity of the second trabecula, right side
24	Junction point of the second trabecula and the sternum body, right side
25	Medial extremity of the dorsolateral process of coracoidal articular facet, right side
26	Ventral extremity of the dorsolateral process of coracoidal articular facet, right side
27	Lateral extremity of the dorsolateral process of coracoidal articular facet, right side
28	Ventral extremity of the lateral crest, right side

**Table 4:** Definition of the landmarks of the coracoid used in the geometric morphometric analysis. See Figs. 2E and 2F for landmark position on the coracoid.

Landmarks	Definition
1	Lateral extremity of the mediodistal angle
2	Medial extremity of the sternal facet
3	Medial extremity of the sternocorocoidal processus
4	Proximal extremity of the glenoid facet
5	Proximal extremity of the procoracoid
6	Apex of the acromiun
7	Apex of the brachial tuberosity
8	Distal extremity of the procoracoid

**Table 5:** Definition of the landmarks of the scapula used in the geometric morphometric analysis. See Figs. 2G and 2H for landmark position on the scapula.

Landmarks	Definition
1	Medial extremity of the acromium
2	Lateral extremity of the acromium
3	Apex of the Tuberculum coracoideum
4	Dorsal extremity of the glenoid facet
5	Ventral extremity of the glenoid facet
6	Apex of the scapular tubercle
7	Caudoventral extremity of the blade of the scapula

**Table 6:** Definition of the landmarks of the humerus used in the geometric morphometric analysis. See Figs. 2I and 2J for landmark position on the humerus.

Landmarks	Definition
1	Distal point of the beginning of the central pneumatic fossa
2	End of the margo caudalis
3	Apex of the bicipital crest
4	Beginning of the dorsal crus
5	Beginning of the ventral crus
6	End of the ventral crus
7	Ventral extremity of the ligamental groove
8	Lateral extremity of the capital groove
9	Proximal extremity of the capital groove
10	Medial extremity of the capital groove
11	Ventral extremity of the head of the humerus
12	Apex of the head of the humerus
13	Dorsal extremity of the head of the humerus
14	Proximal extremity of the deltoid crest
15	Apex of the deltoid crest
16	Distal extremity of the deltoid crest
17	Proximal extremity of the entipocondyle, medial view
18	Apex of the entipocondyle, medial view
19	Distal extremity of the external epicondyle
20	Apex of the ventral condyle
21	Distal point of the medial epicondyle
22	Proximal point of the dorsal condyle, lateral view

### 3D sliding-landmark procedures

The 3D sliding landmark procedure (Bardua et al., 2019; Bookstein, 1997; Gunz, Mitteroecker & Bookstein, 2005) was used in this study. In this procedure, sliding landmarks are transformed into spatially homologous landmarks that can be used to compare shapes. They will slide along curves that are predefined on each surface. This operation is performed using the Morpho package in R (v3.5.0) (Schlager, 2017; Schlager, Jefferis & Ian, 2019). Curves and surface sliding-landmarks are projected from the template onto each specimen for each data set. In this step, each new specimen is only defined by its landmarks and semi landmarks on curves. Next, the surface sliding-landmarks are projected onto the predefine curves and the surface of the new specimen using a template. Finally, spline relaxation was performed minimizing the bending energy criterion.

### Generalized procrustes superimposition

Generalized Procrustes Superimposition or GPA (Rohlf & Slice, 1990) allows the comparison of an object’s shape by removing size, orientation, and position relatively to the origin of coordinate system. We computed the first step which was an operation of translation of all the objects, allowing the superimposition on their center of gravity. The second step was an operation of normalization; all the objects were scaled and end up having the same scale. During this operation, all the coordinates of each object were divided by the centroid size which was the square root of the summed squared distances of each landmark to the centroid (Bookstein, 1997). Finally, each conformation was rotated by minimizing the summed square distances between all the landmarks. We performed the GPA using the function ‘‘gpagen’’ in Geomorph R package (Adams & Otárola-Castillo, 2013).

After superimposition, each object was defined by Procrustes coordinates and rescaled. Thus, differences in conformation or objects shape could be studied and were simply represented by changes in the proportion of structures. After this operation has been performed for each data set, the landmarks of all specimens were comparable.

### Statistical analysis

All the statistical analyses below were done in R (v.3.5.0; R Core Team, 2018).

### Principal component analysis

In order to explore the distribution of the specimens in the morphological space (morphospace) and to reduce the number of dimension of our dataset, we performed a principal component analysis (PCA) using the function plotTangentSpace of “geomorph” package in R (Adams & Otárola-Castillo, 2013).

### Difference of bone shape depending of bone texture

We wanted to compare, for each bone, the external appearance as a proxy for deformation due to preparation. Each bone was categorized depending on its external appearance, from oily to powdery. We created three categories: oily for yellow and shiny bones meaning lot of fat remained, powdery for bones that are very white and dusty representing little fat and neutral for the other bones. We tested for shape differences depending on these qualitative categories using a multivariate analysis of variance (MANOVA) on the principal component scores (PC) accounting for 95% of the overall variance of each bone (furcula: 10 PCs representing 95.5%, sternum: 11 PCs representing 95.7%, coracoid: 22 PCs representing 95.5%, scapula: 18 PCs representing 95.4% and humerus: 23 PCs representing 95.1% of the overall variance).

### Visualizing shape similarities using a neighbor joining tree

We computed neighbor joining trees on the Euclidean distances using at least 95% of the overall variance in order to obtain unrooted trees.

### Quantification of asymmetry to assess the impact of bone preparation using *t*-test

In order to quantify the preparation effect, we tested the presence of asymmetry using a paired student test comparing right and left parts of the bones (Kharlamova et al., 2010). We used the *t*-test function in basic package in R. In the same way, we compared symmetrized and non-symmetrized shapes.

### Quantification of disparity for each bone shape

We also calculated morphological disparity of each bone in both datasets thanks to the D index which give us a numerical value showing how different bones are between each other using the morphol.disparity function in “geomorph” package in R (Zelditch et al., 2004).

### Assessing a possible effect of bone preparation on interspecific morphological studies

Finally, we performed a PCA and disparity analyses on the interspecific data set in order to compare it to the intraspecific variability. It allows to assess a possible effect of bone preparation on interspecific morphological studies. If the impact of bone preparation is low, we expect to see a clustering of all the *C. coturnix* in the same part of the morphospace, whereas the other species should occupy a larger part of the morphospace. We also expect that the disparity of *C. coturnix* will be lower than those of all the other species combined.

## Results

### Intraspecific level

#### Shape differences depending on texture or color

**Figure 3:**
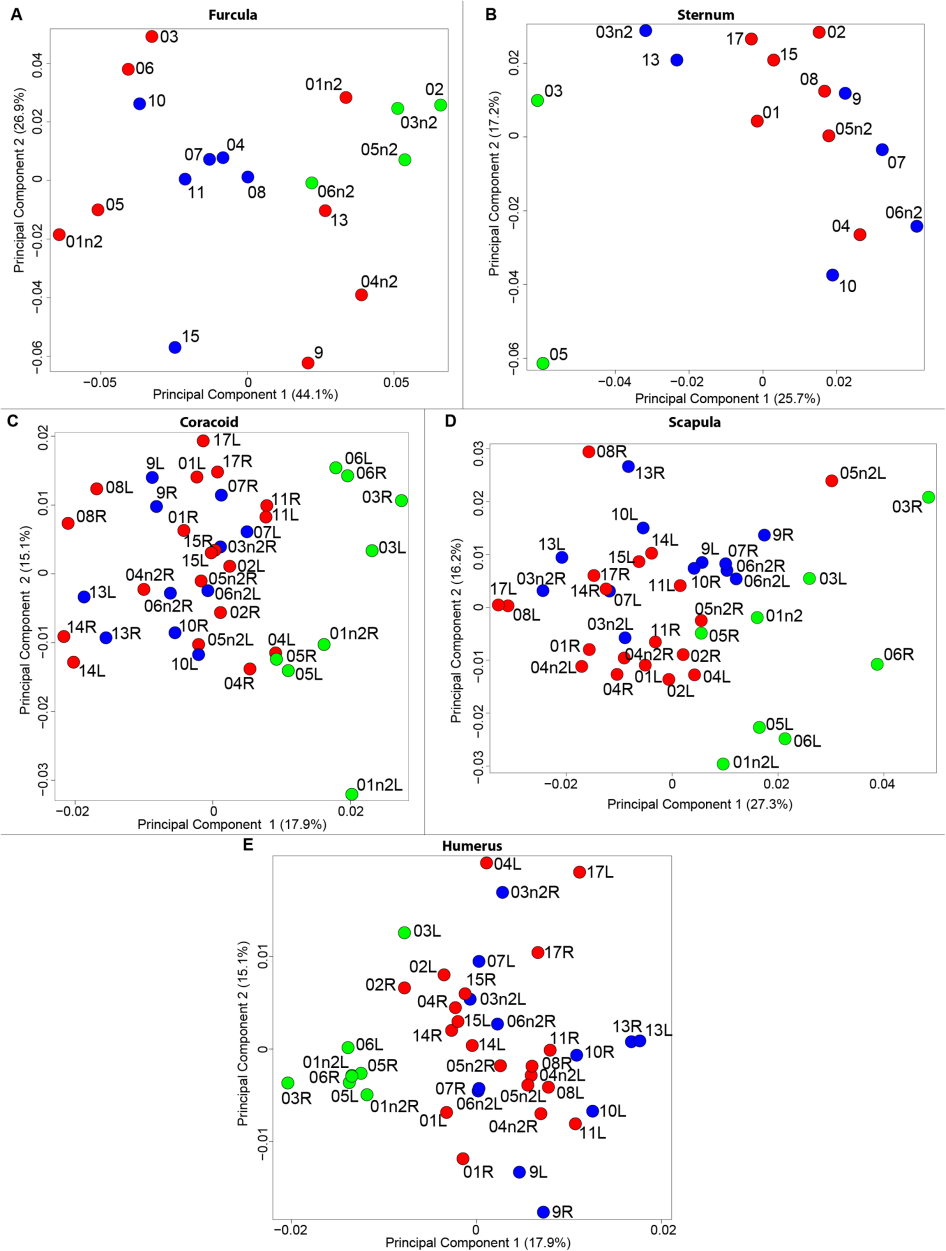
Overview of the results of Principal Component Analyses (PCA) performed on quails bone shapes. Colors represent bone texture: green is for powdery bones, blue is for neutral bones and red is for oily bones. Each individual is identified thanks to a unique code, for paired bones we add the information of the side: L left and R right. (A) Furcula, (B) sternum, (C) coracoid, (D) scapula, (E) humerus.

The results of the MANOVAs showed that powdery bones are significantly morphologically different from neutral and oily bones (*p*-value below 0.01; Fig. 3; Table 7). Powdery bones in comparison to neutral and oily ones are characterized by furculae with narrower clavicles, sterna with dorsolateral and caudolateral processes that are more distant from the central part, coracoids with a thinner shaft, scapulae with a thinner blade and humeri with a more gracile shaft. No shape differences were found between oily and neutral bones.

**Table 7:** Results of the MANOVAs testing for shape differences depending of the texture for each bone. Significant differences are indicated in bold.

Bone PC1 scores	Oily	Neutral	Powdery
Furcula	x	x	**0.01**
Sternum	x	x	**<0.01**
Coracoid	x	x	**<0.01**
Scapula	x	x	**<0.01**
Humerus	x	x	**<0.01**

#### Furcula

**Figure 4:**
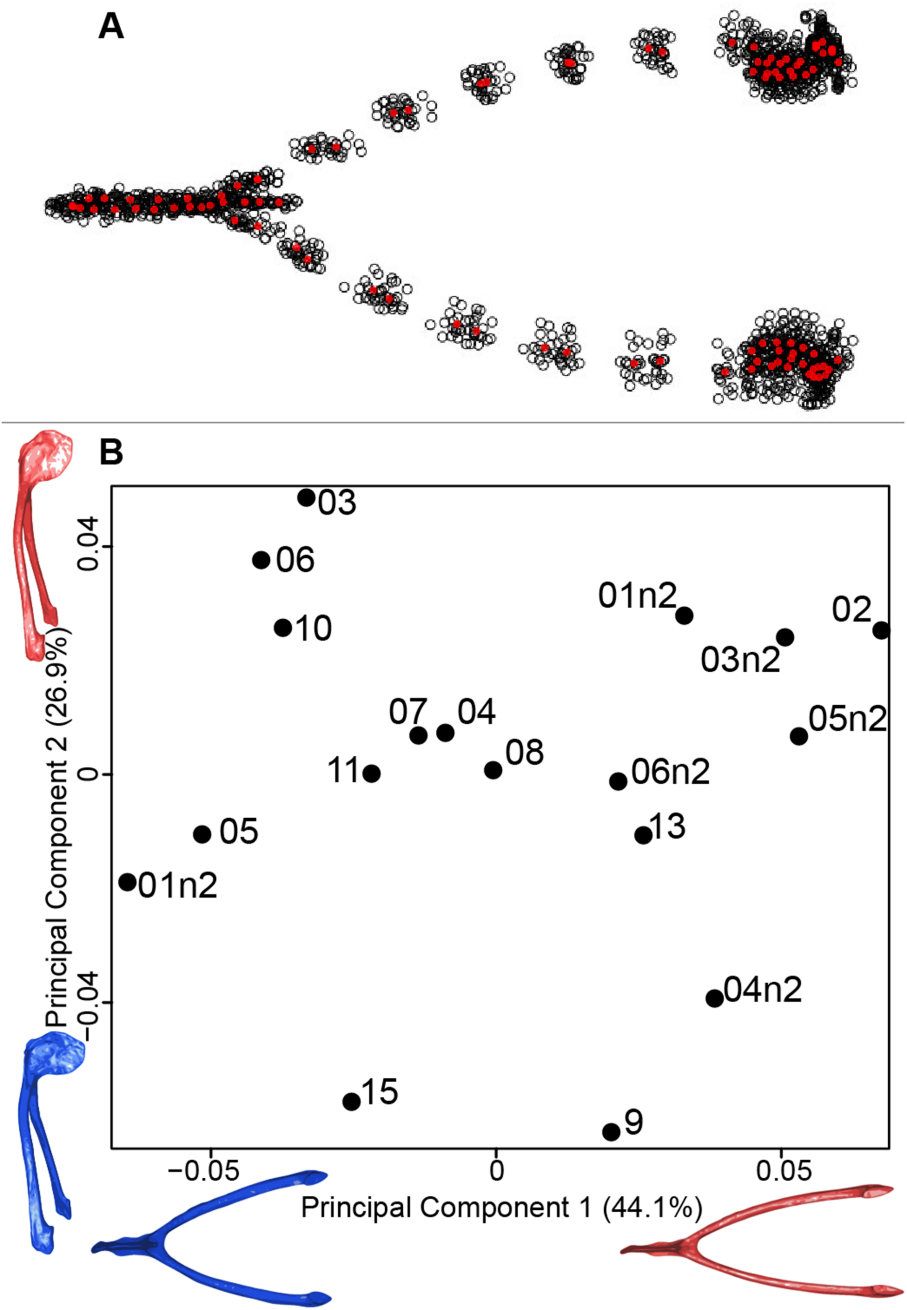
Overview of the analyses of the quail furculae. (A) Consensus shape plot of the quail furculae. Consensus shape is shown in red points, all shapes observed are in black points. (B) Principal Component Analysis performed on quail furcula shapes. Maximum theoretical shapes are shown in red and minimum theoretical shapes are in blue. Each individual is identified thanks to a unique code.

We computed the consensus shape of the furcula. The points on each side were very dispersed which means that there is considerable shape variation in the furculae (Fig. 4A). The four first axes of the intraspecific PCA explained 83.5% of the total variance (PC1 = 44.1%, PC2 = 26.9%, PC3 = 7.4% and PC4 = 5.1%; Fig. 4B). Two types of shapes were distinguished along the first axis. The negative axis was represented by a furcula with the clavicles being more distant from one another and a rounded caudally oriented symphysis. On the contrary, narrow clavicles and elongated dorsally oriented symphysis were situated towards the positive side of the axis.

#### Sternum

**Figure 5:**
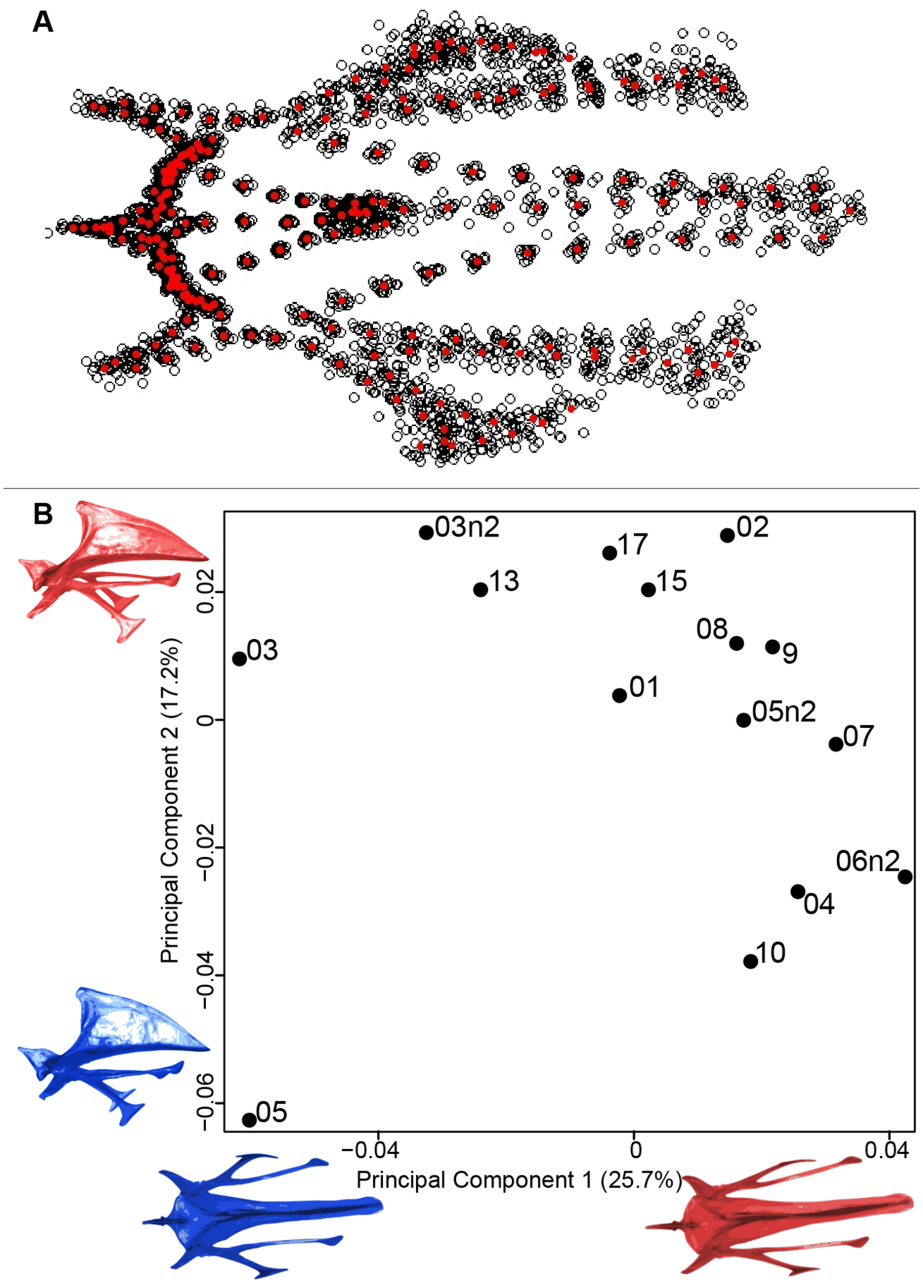
Overview of the analyses of the quail sternums. (A) Consensus shape plot of the quail sternum. Consensus shape is shown in red points, all shapes observed are in black points. (B) Principal Component Analysis performed on quail sternum shapes. Maximum theoretical shapes are shown in red and minimum theoretical shapes are in blue. Each individual is identified thanks to a unique code.

We found the same pattern for the sternum as observed for the furcula (Fig. 5A). Thin parts on each side were very variable in orientation and shape. However, both the center part and the keel, showed little deformation. The four first axes of the PCA explained 65.3% of the total variance (PC1 = 25.7%, PC2 = 17.2%, PC3 = 12.0% and PC4 = 8.7%; Fig. 5B). Two types of shapes were distinguished along the first axis. The negative part was represented by a sternum with dorsolateral and caudolateral processes more distant from the central part of the sternum. The second axis showed differences in the anterior part of the sternum with the coracoid joint and the craniolateral process which were more prominent on the negative part of the axis compared to the positive part.

#### Coracoids

**Figure 6:**
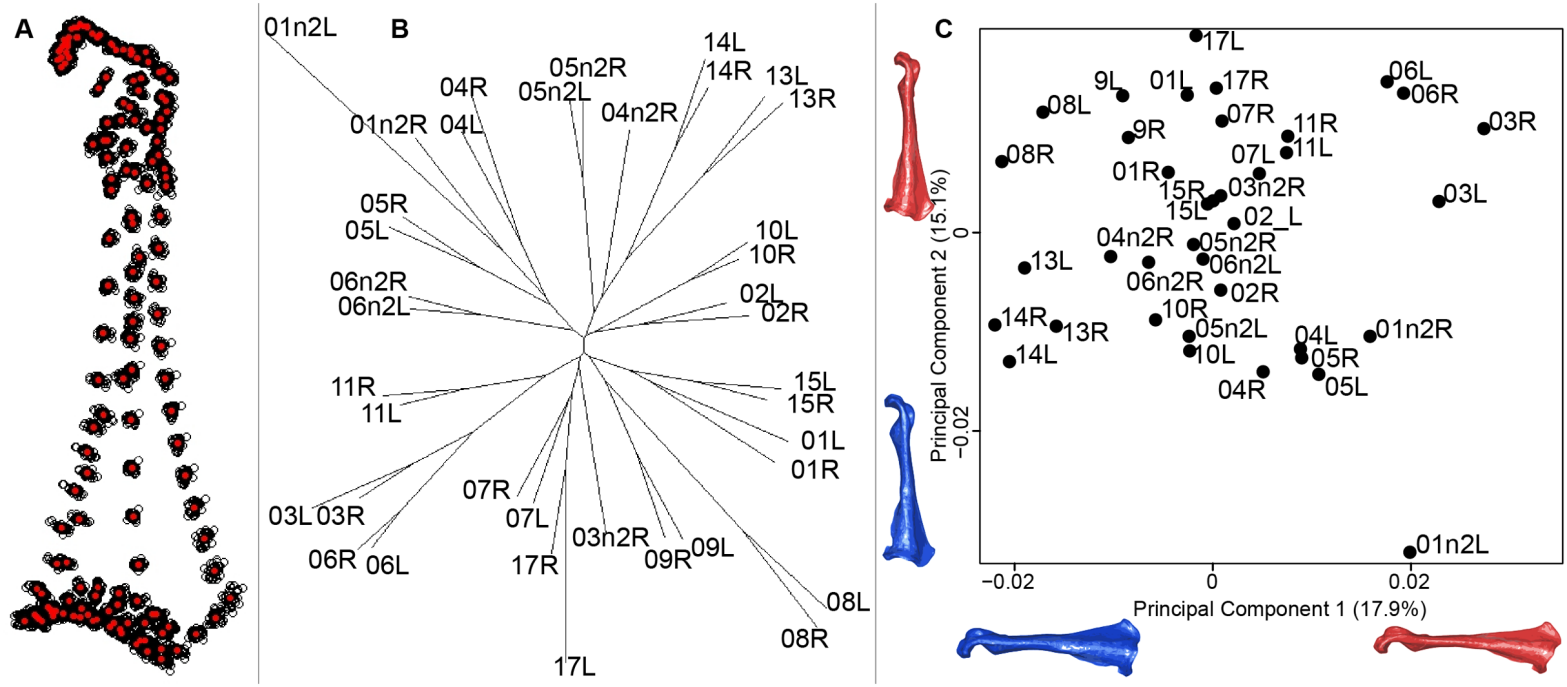
Overview of the analyses of the quail coracoids. (A) Consensus shape plot of the quail coracoids. Consensus shape is shown in red points, all shapes observed are in black points. (B) Quail coracoid shapes neighbor joining tree. Each individual is identified thanks to a unique code, L: left side and R: right side. (C) Principal Component Analysis performed on quail coracoid shapes. Maximum theoretical shapes are shown in red and minimum theoretical shapes are in blue.

For the coracoid bone, which is a paired bone, the consensus shape showed that all the landmarks overlapped (Fig. 6A). This was confirmed by the fact that all right and left coracoids were each other’s closest neighbors in the neighbor joining trees (Fig. 6B). The four first axes of the PCA explained 54.3% of the total variance (PC1 = 17.9%, PC2 = 15.1%, PC3 = 12.0% and PC4 = 9.3%; Fig. 6C). Two types of shapes could be distinguished along the first axis. The positive part was represented by a coracoid with angular sternocoracoidal process. The second axis showed differences on the anterior part of the coracoid with the acromion and the clavicle facet being more prominent on the positive part of the axis than on the negative part.

#### Scapula

**Figure 7:**
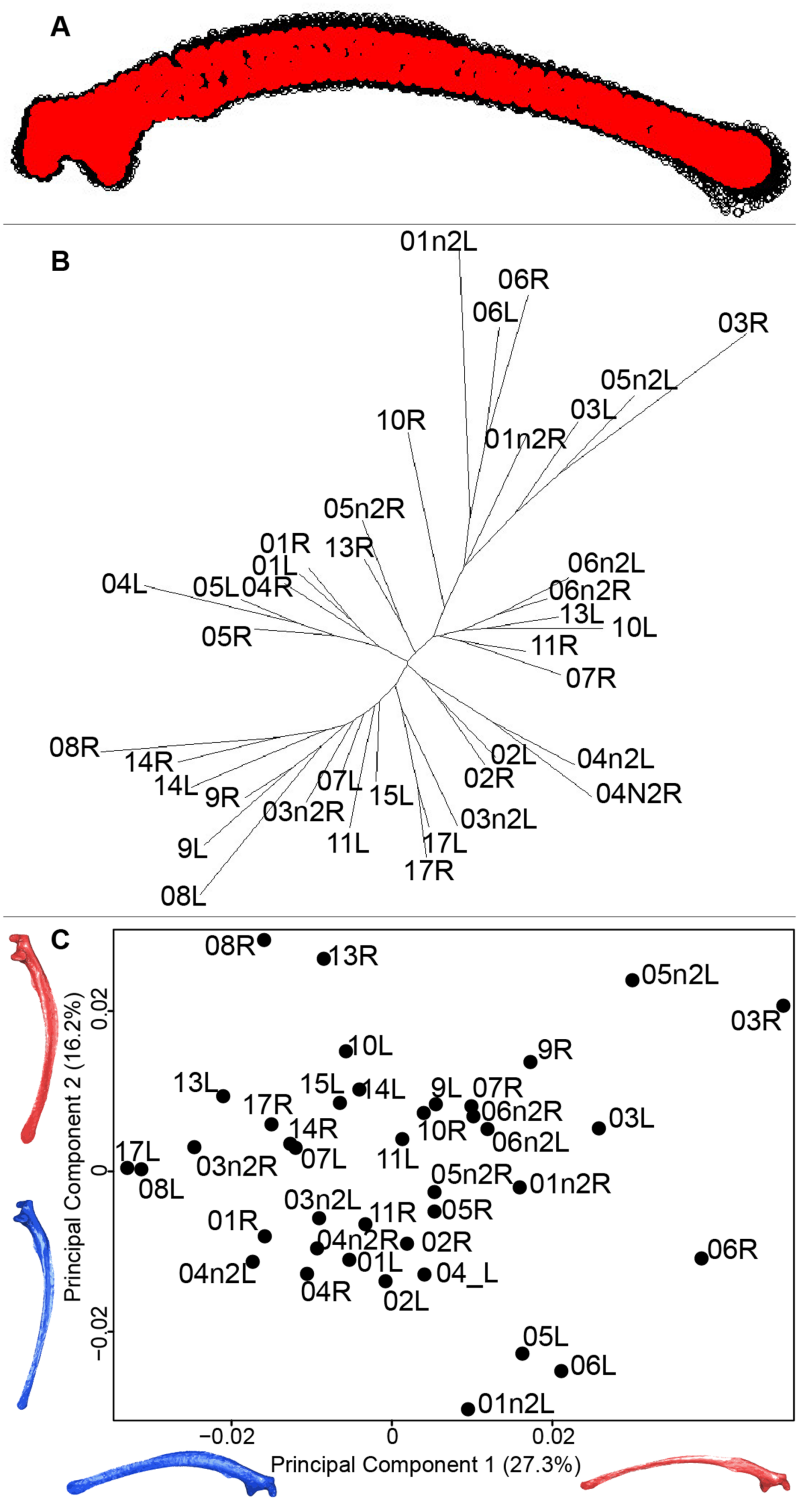
Overview of the analyses of the quail scapulae. (A) Consensus shape plot of the quail scapulae. Consensus shape is shown in red points, all shapes observed are in black points. (B) Quail scapula shapes neighbor joining tree. Each individual is identified thanks to a unique code, L: left side and R: right side. (C) Principal Component Analysis performed on quail scapula shapes. Maximum theoretical shapes are shown in red and minimum theoretical shapes are in blue.

Scapula consensus shape showed that all the landmarks overlapped (Fig. 7A). Yet, not all right and left scapulae were each other’s closest neighbors in neighbor joining trees (Fig. 7B). The four first axes of the PCA explained 67.8% of the total variance (PC1 = 27.3%, PC2 = 16.2%, PC3 = 14.2% and PC4 = 10.1%; Fig. 7C). Along the first axis, the positive part was represented by a gracile scapula with the anterior part of the blade being enlarged. The second axis showed differences on the global robustness of the blade on the positive part of the axis and a more gracile and curved blade on the negative part.

#### Humerus

**Figure 8:**
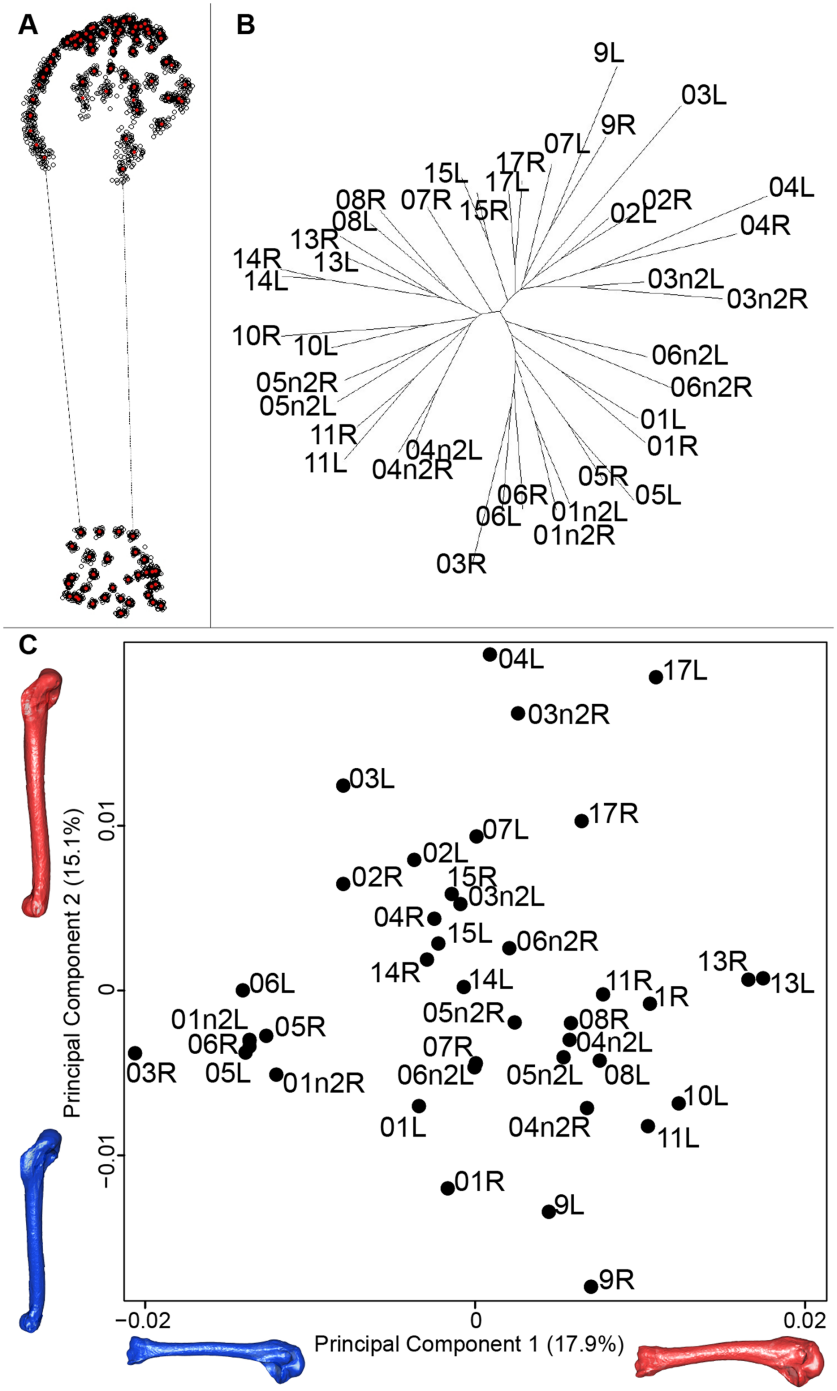
Overview of the analyses of the quail humeri. (A) Consensus shape plot of the quail humeri. Consensus shape is shown in red points, all shapes observed are in black points. (B) Quail humerus shapes neighbor joining tree. Each individual is identified thanks to a unique code, L: left side and R: right side. (C) Principal Component Analysis performed on quail humerus shapes. Maximum theoretical shapes are shown in red and minimum theoretical shapes are in blue.

For the humerus, the consensus shape showed that all the landmarks overlapped (Fig. 8A). This seemed to be congruent with the neighbor joining tree where almost all right and left humeri were each other’s closest neighbors (Fig. 8B). The four first axes of the PCA explained 51.7% of the total variance (PC1 = 17.9%, PC2 = 15.1%, PC3 = 10.2% and PC4 = 8.5%; Fig. 8C). The positive part was represented by a robust humerus with a large shaft and articulation. In contrast, gracile humeri with long and thin shaft were associated with the negative part of the axis. The second axis highlighted a difference in the head length on the anterior part of the humerus with a longer head at the negative part of the axis.

#### Disparity and symmetry

Unpaired bones, furcula and sternum, had a higher disparity than paired bones (Table 8). Symmetry tests showed that the bones have different patterns of symmetry (Table 9). Unpaired bones, such as the furcula and the sternum, seemed to be less symmetrical than paired bones such as the coracoid, scapula and the humerus. Among the paired bones, the results showed that the sternum seemed to be more asymmetrical than the furcula. These symmetry test results were congruent with the disparity tests.

**Table 8:** Morphological disparity index for each bone (×100,000).

Bone/Sampling	Intraspecific level	Interspecific level
Furcula	333	2024
Sternum	822	6382
Coracoid	83	699
Scapula	95	301
Humerus	38	187

**Table 9:** Results of the symmetry tests performed on each bone for the intraspecific dataset. Significant differences are indicated in bold.

Bone	Student *T* value	Student test *P*-value
Furcula	**−29.1**	**<0.01**
Sternum	**−48.3**	**<0.01**
Coracoid	−1.4	0.16
Scapula	<0.001	1
Humerus	<0.001	1

### Interspecific level analyses to assess the impact of bone deformation in a broader context

#### Furcula

**Figure 9:**
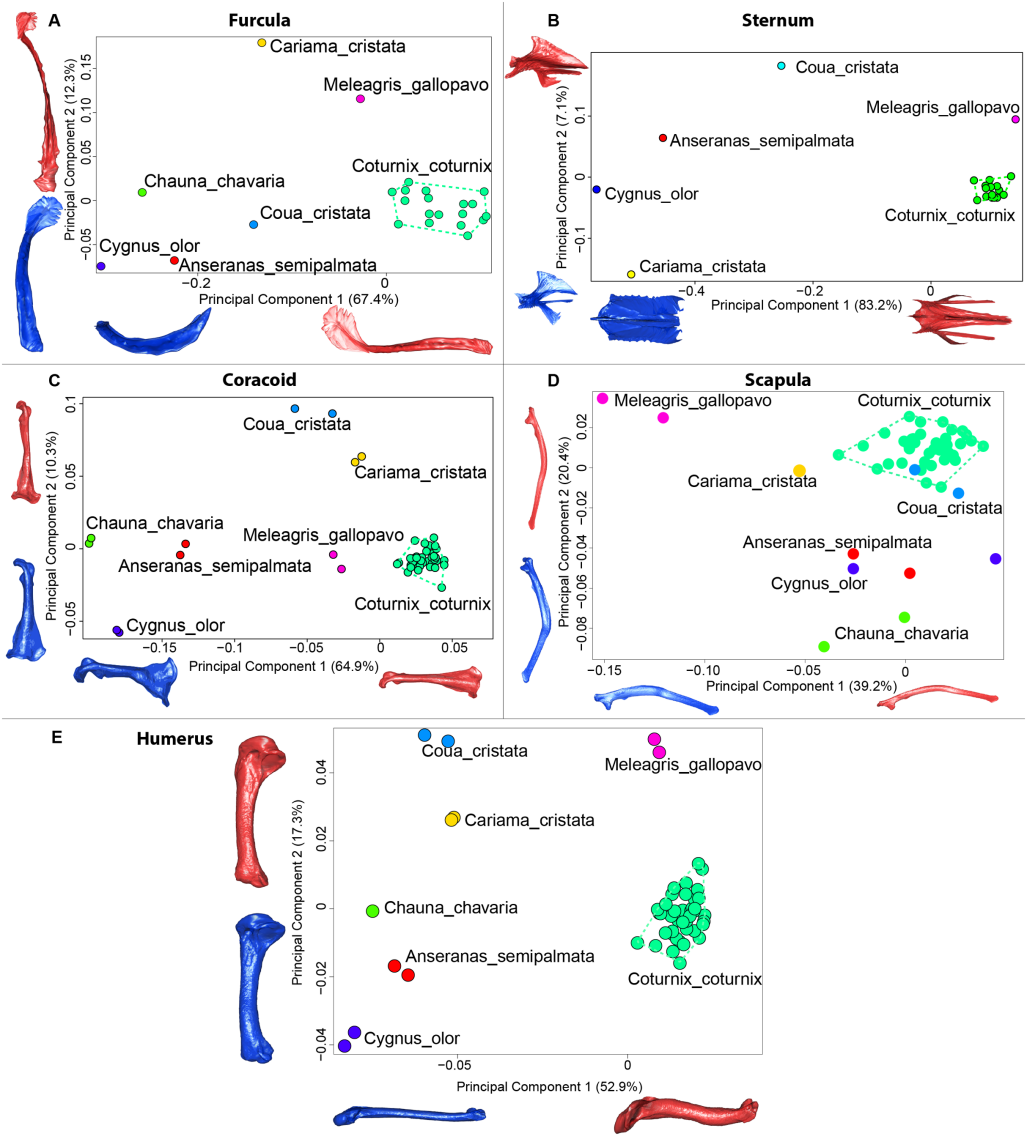
Overview of the results of Principal Component Analyses (PCA) performed on interspecific dataset of bone shapes. Colors represent each species. *C.coturnix* specimens are linked together. (A) furcula, (B) sternum, (C) coracoid, (D) scapula, (E) humerus. Maximum theoretical shapes are shown in red and minimum theoretical shapes are shown in blue.

The first four axes of the interspecific PCA explained 91.8% of the total variance (PC1 = 67.4%; PC2 = 12.3%; PC3 = 8.5%; PC4 = 3.6%; Fig. 9A). The quail specimens grouped together whereas the other species were spread in the morphospace. The disparity calculation showed a larger disparity between species than among quails (Table 8).

#### Sternum

The interspecific PCA four first axes explained 95.9% of the total variance (PC1 = 83.2%; PC2 = 7.1%; PC3 = 3.5%; PC4 = 2.1%; Fig. 9B). Quail specimens grouped together whereas the other species were widespread in the morphospace. The disparity calculation showed an eight times larger disparity at the interspecific level compared to the intraspecific level (Table 8).

#### Coracoid

The four first axes of the PCA computed on the sternum shapes explained 86.6% of the total variance (PC1 = 64.9%, PC2 = 10.3%, PC3 = 6.8% and PC4 = 4.6%; Fig. 9C). In this morphospace, all the quails were packed and well differentiated from other species. Again, disparity calculations supported this result (Table 8).

#### Scapula

The four first axes of the PCA computed on the coracoid shapes explained 79.8% of the total variance (PC1 = 39.2%, PC2 = 20.4%, PC3 = 12.9% and PC4 = 7.3%; Fig. 9D). Quails were clustered together, yet, *Coua cristata* overlapped with the quails on the first two axes. The disparity calculation confirmed that there was less disparity among quails than at the interspecific level (Table 8).

#### Humerus

The four first axes of the PCA computed on the scapula shapes explained 81.7% of the total variance (PC1 = 52.9%, PC2 = 17.3%, PC3 = 7.5% and PC4 = 4.0%; Fig. 9E). Quail bones clustered together and were well separated from other species, which corresponds to the disparity estimates (Table 8).

## Discussion

The preparation process is an obligatory step in the preparation of bones for collections. It is, however, important to be able to quantify potential effects of preparation on the morphology of the treated bone as this may impact subsequent comparative studies. Some effects have been reported, such as modified microstructure and modification of the chemical composition of the bone (Fernández-Jalvo & Marin-Monfort, 2008; Hahn, Vogel & Delling, 1991; Lemoine, 2011). In practice, it appears to be no specific preparation protocol for bird bones. Yet, birds bones are pneumatic and this characteristic should be taken into account during preparation (Baumel et al., 1993; Fernández-Jalvo & Marin-Monfort, 2008; Novitskaya et al., 2017; Pennycuick, 1967; Ritzen, 1978). Moreover, the preparation protocol with enzymes used for our bones is one of two best protocols studied by Fernández-Jalvo & Marin-Monfort (2008) to avoid physical damage.

### What is the impact of deformation due to preparation on the bone shape at the intraspecific level?

#### Differences in shape depending on the color and texture

The results of the MANOVAs performed on each bone show significant shape differences depending on the texture. The main differences are between powdery bones and other types of bone (Fig. 3; Table 7). Powdery bones appear to have a wider distribution in the morphospace for each bone. Considering extreme bones shapes shown in the PCA for each bone, most of the time the gracile shapes match the powdery bones. This suggests a direct impact on the thickness and the composition of the bone because of the preparation process.

Looking more specifically at the concerned individuals, some individuals have powdery bones for all the paired bones. A oilyness texture is not found on all the bones of the same specimen, which suggest that this characteristic may not be individual-specific. It could be linked to the type of preparation, more specifically to the removal of the fat. Preparators are used to evaluate the fat saturation by looking at the bone texture directly after an obligatory first bath. There are three possibilities during preparation: (1) the fat saturation of the bone looks low and the treatments are stopped; (2) the fat saturation of the bone is still too important so the renewal of this step is decided or (3) the first bath treatment itself may be too aggressive for the bone and texture is already powdery after the initial fat removal step. It is known that the bird furcula is composed of Haversian bone for a large part of the fused part of the clavicle (Cubo et al., 2005; Mitchell et al., 2017; Ponton et al., 2007). This particular bone formation may result in a different reaction when treated with the chemicals used in the preparation protocol (Lemoine & Guilminot, 2009). For this reason, preparation protocols have to be adapted to the specific bones (Hahn, Vogel & Delling, 1991). Because all individuals and bones may differ in internal composition, length, width, weight and thickness, using the same quantity of chemicals or the same time of processing for all bones could impact the bone. The external appearance of the bone appears to be a good indicator of the impact of preparation and as such a good proxy for preparation deformation. It would be interesting in future studies to perform histological analyses to be able to detect the effect of chemicals on the preparation of the bones.

#### Furcula

The analysis of the furcular shape shows that the main shape modifications occur on the clavicles and their symphysis. Considering the results of the PCA and shape differences depending on the texture, the deformation appears to result in a flatter furcula with narrower and straighter clavicles and with an elongated and more dorsally oriented symphysis (Fig. 4). These shape modifications could be explained by a modification of the Haversian bone, which is specifically located in this area of the furcula. Indeed, furcula bone composition is known to be different from the other bird bones (Mitchell et al., 2017). Furthermore, wing beats during locomotion have been shown to induce cyclic deformations, with bone remodeling replacing damaged bone with Haversian bone (Ponton et al., 2007). This bone type seems more likely to be affected by the chemical preparation process compared to the non-Haversian bone.

#### Sternum

The main parts of the sternum shape affected by preparation are the lateral processes, the thicker parts of the sternum which appear more distal from the central part (Fig. 5). The central part of the sternum has a protection function and provides support for the carina. This part of the sternum is thick and robust to hold the pectoral muscles and to withstand their force (Baumel et al., 1993; Harvey, Kaiser & Rosenberg, 1969). The cranial and central part is involved into the coracoid joint area, it functional constraint could explain the light amount of deformation. The lateral thin parts of the sternum are inter-connected with fasciae and aponeuroses of the flat oblique abdominal muscle (Goslow, Dial & Jenkins, 1990). Moreover, these abdominal muscle forces may deform the bone during wingbeats to keep the unity of the trunk (Jenkins, Dial & Goslow, 1988). Jenkins, Dial & Goslow (1988) showed that the sternum also exhibits cyclical movements with each wingbeat. During down-stroke, the sternum ascends and retracts caudodorsally, and then during the subsequent upstroke it descends and protracts cranio-ventrally. As in the furcula, flexible parts of the sternum involved in wingbeats seem to be more easily affected by the preparation process.

#### Coracoid

Coracoid bones display less shape variation than unpaired bones. The main shape modification seems to be the gracile conformation of the bone. The shaft is sharper, the distal part is sharp-edged and the proximal part is more curved. These deformations look like a slight contraction of the whole bone on itself. Coracoids have an important function during flight, as they acts as a pulley for the pectoral muscles, which are the biggest muscles involved in the wing upstroke. Coracoids have to be robust enough to support and transmit muscles forces without deforming (Beaufrère, 2009; Nesbitt et al., 2009). Its crucial role in force transmission could be a strong constraint on both shape and robustness (George & Berger, 1966; Shufeldt, 1901, 1909). This result seems to be confirmed by the neighbor joining tree, showing that both right and left coracoids are well paired for each individual (Fig. 6). This result supports the hypothesis of strong solidity of this bone (George & Berger, 1966; Gordon et al., 2008).

#### Scapula

The neighbor joining tree performed on the shape data of the scapula shows some morphological variation between the right and left bones for each individual. Natural asymmetry is not expected to be higher within individuals than between individuals, thus, these differences could be due to the preparation process. This result is supported by the wide distribution in the morphospace, especially on the positive part of the first axis which is characterized by a gracile and low scapula (Fig. 7). This suggests that these morphologies may not be due to natural asymmetries but more likely due to the preparation process (Hahn, Vogel & Delling, 1991; Lemoine, 2011).

#### Humerus

In contrast to the results obtain for the scapula, the neighbor joining tree of the humeri shows that left and right bones belonging to the same individual cluster together. This suggests that the preparation process may have less impact on the humerus. Looking at the PCA, a group of bones seems more isolated from the others. Their shape is gracile, the deltoid crest is less prominent and the distal extremity is less robust (Fig. 8). As for the scapula, extreme humerus bone shapes have a more gracile morphology than the mean bone shapes. Moreover, the humerus is known to be not significantly loaded in direct tension or compression, which implies no particular ossification or solidification of this bone (Pennycuick, 1967). Again, it suggests a non-natural deformation and thus could be due to preparation process affecting the thickness of the whole bone (Hahn, Vogel & Delling, 1991; Lemoine, 2011).

In general, powdery paired bones are more gracile than neutral and oily bones. It seems that the last step of the preparation protocol, the fat removal which can be repeated several times, is the main factor causing bone shape deformation.

#### Disparity and asymmetry

We observed that unpaired bones display a greater disparity than paired ones and the same pattern is found in interspecific analyses (Table 8). This could mean that unpaired bones are more easily deformed by preparation than paired bones. This could be explained by two factors: (1) paired bones can easily be dried in a specific position. For unpaired bones, the most convenient method is to put it on its side. Thus, this position can induce a morphological deformation only on one side due to the fact that the bones have to support their own weight. This way of drying can lead the bone to have a directional drying asymmetry; (2) all vertebrates display a bilateral symmetry, yet are not perfectly symmetric. Many factors can impact symmetry including lateralisation (Galatius & Jespersen, 2006; Klingenberg, 2003; Mays, Steele & Ford, 1999; Palmer, 2004). This phenomenon should, however, impact paired and unpaired bones similarly. However, the symmetry tests shows a significant differences between right and left sides for unpaired bones, such as furculae and especially sterna, whereas the differences are not significant for paired bones. Given that one side is always significantly different from the other one this suggests an impact of the drying process on bone asymmetry.

### What is the impact of deformation due to preparation on shape analysis at the interspecific level?

The interspecific dataset demonstrates that, despite the large morphological disparity observed within the quail dataset, analyses conducted at an interspecific level are not impacted by the effect of bone preparation (Table 8). It suggests that, even if there are some deformations due to the preparation protocol, at an intraspecific dataset level of analyses, these deformations are too small to be significant.

## Conclusions

In summary, it appears that flexible bones and bones with thin parts such as the blades of the sternum and scapula are more likely deformed by the preparation process. However, the central part of the sternum and the keel which provide protection and have large muscle insertions or the coracoid with its robust pulley function are not deformed. Symmetry tests show that shape variations cannot be natural because they are located mainly on unpaired bones and are not equally distributed between the two sides of the bone. Thus, the drying process could induce some deformations on unpaired bones. Moreover, for paired bones, the more gracile bone shape with a powdery texture appeared to be a direct consequence of the preparation process. We showed that these preparation deformations can influence intraspecific analysis and lead to functional erroneous conclusions, especially when studying the effect of symmetry on bones. Finally, these deformations due to the preparation have little effect at the interspecific level. This study highlights the importance of carefully selecting preparation methods in order to avoid physical damage that could impact the shape of the treated bones. To more accurately understand the effect of preparation on the deformation of bones, future studies need to be done comparing X-ray computed tomography of specimens before and after preparation.

## Additional Information and Declarations

### Data Availability

The following information was supplied regarding data availability: The 3D data are available at Morphosource: https://www.morphosource.org/Detail/ProjectDetail/Show/project_id/824.
